# Enhancement of Activity and Development of Low Pt Content Electrocatalysts for Oxygen Reduction Reaction in Acid Media †

**DOI:** 10.3390/molecules26175147

**Published:** 2021-08-25

**Authors:** Aldona Kostuch, Iwona A. Rutkowska, Beata Dembinska, Anna Wadas, Enrico Negro, Keti Vezzù, Vito Di Noto, Pawel J. Kulesza

**Affiliations:** 1 Faculty of Chemistry, University of Warsaw, Pasteura 1, PL-02-093 Warsaw, Poland; aldonakostuch@gmail.com (A.K.); ilinek@chem.uw.edu.pl (I.A.R.); bbaranowska@chem.uw.edu.pl (B.D.); awadas@chem.uw.edu.pl (A.W.); 2 Department of Industrial Engineering, Università degli Studi di Padova, Via Marzolo 1, 35131 Padova, Italy; enrico.negro@unipd.it (E.N.); keti.vezzu@gmail.com (K.V.); vito.dinoto@unipd.it (V.D.N.)

**Keywords:** oxygen reduction, electrocatalysis, low Pt loading, sub-stoichiometric metal oxides, doping and functionalization of carbon carriers, Pt alloys

## Abstract

Platinum is a main catalyst for the electroreduction of oxygen, a reaction of primary importance to the technology of low-temperature fuel cells. Due to the high cost of platinum, there is a need to significantly lower its loadings at interfaces. However, then O_2_-reduction often proceeds at a less positive potential, and produces higher amounts of undesirable H_2_O_2_-intermediate. Hybrid supports, which utilize metal oxides (e.g., CeO_2_, WO_3_, Ta_2_O_5_, Nb_2_O_5_, and ZrO_2_), stabilize Pt and carbon nanostructures and diminish their corrosion while exhibiting high activity toward the four-electron (most efficient) reduction in oxygen. Porosity of carbon supports facilitates dispersion and stability of Pt nanoparticles. Alternatively, the Pt-based bi- and multi-metallic catalysts, including PtM alloys or M-core/Pt-shell nanostructures, where M stands for certain transition metals (e.g., Au, Co, Cu, Ni, and Fe), can be considered. The catalytic efficiency depends on geometric (decrease in Pt–Pt bond distances) and electronic (increase in d-electron vacancy in Pt) factors, in addition to possible metal–support interactions and interfacial structural changes affecting adsorption and activation of O_2_-molecules. Despite the stabilization of carbons, doping with heteroatoms, such as sulfur, nitrogen, phosphorus, and boron results in the formation of catalytically active centers. Thus, the useful catalysts are likely to be multi-component and multi-functional.

## 1. Introduction

The rapid growth of energy consumption and the necessity to reduce greenhouse gas emissions associated with energy production have been promoting a search for alternative energy sources. Fuel cells (FCs) are one of the most important solutions in this respect due to their high electrical efficiency, low pollution level, silent operation, and fuel diversity [1]. During the electrochemical reactions occurring at the FCs electrodes (anode and cathode), the chemical energy of a fuel and an oxidant are directly converted into electrical energy. Among various types of FCs, the proton exchange membrane fuel cell (PEMFC) technology is one of the most promising approaches for energy conversion in automotive applications [2]. Despite the use of expensive noble metal electrocatalysts [3,4], the sluggish kinetics of the oxygen reduction reaction (ORR) in acid media, and formation of the undesirable hydrogen peroxide intermediate are still the main drawbacks, on top of the stability problems, when it comes to the widespread commercialization of PEMFC devices.

In acid media, the ORR can proceed according to the four-electron or two-electron mechanisms, each characterized by distinct thermodynamic potentials (E°, expressed vs. Reversible Hydrogen Electrode, RHE, at standard conditions and 25 °C):

direct four-electron reduction:

O_2_ + 4H^+^ + 4e^−^ → 2H_2_O   E° = 1.230 V (1)

indirect two-step reduction:

O_2_ + 2H^+^ + 2e^−^ → H_2_O_2_   E° = 0.680 V (2)

followed by reduction of hydrogen peroxide

H_2_O_2_ + 2H^+^ + 2e^−^ → 2H_2_O   E° = 1.776 V (3a)

or its chemical decomposition:

2H_2_O_2_ → 2H_2_O + O_2_   E° = 1.776 V (3b)

The four-electron mechanism is energetically more favored with respect to potential applications in the low-temperature fuel cell technology. The progress in this subject is greatly hindered by the high cost and scarcity of the state-of-the-art platinum-based materials which are regarded as the most effective cathode catalysts. Therefore, most of the current research studies have been devoted to the optimization of active centers and maximization of their utilization, which should allow the lowering of the cathode Pt loadings without loss of performance and durability [5,6,7,8]. Unfortunately, the problem of electrochemical stability and the danger of generation of higher quantities of the undesirable hydrogen peroxide intermediate may become even more serious in the case of systems utilizing lower amounts of the Pt catalyst [9].

The present study addresses different approaches and concepts toward the more efficient utilization of the platinum catalytic nanostructures during ORR. Although the exact numbers are not generally defined, depending on the type of experiment: diagnostic (voltammetric, including rotating disk methodology) or with use of fuel cell, application of low Pt contents would concern amounts typically below (sometimes even much lower than) 15–20 or 150–200 µg cm^−2^, respectively. Among important strategies is hybridization, activation, and stabilization of carbon-supported Pt catalysts by functionalization through admixing with certain nanostructured and typically substoichiometric metal oxides. Special attention is paid to application of the bi- or multi-metallic Pt-based alloys in their various forms and structures. Additionally, doping of carbon carriers with heteroatoms would produce surface functional groups and active centers capable of not only improving the catalysts’ performance, but also affecting their stability. Among other important issues are such features as porosity, hydrophilicity, and degree of graphitization of carbon components, in addition to the existence of metal–support interactions, high electrochemical active surface area, electronic structure of interfacial Pt, and the feasibility of adsorptive or activating interactions with oxygen molecules.

## 2. Pt-Metal Oxides (Pt-MO) Hybrid Catalysts

Recent studies demonstrate that the rational design of a catalytic system is based on nanostructures of platinum, or carbon-supported platinum (Pt/C), and metal oxide (MO). By combining the catalytic properties of both components, such hybrid Pt-MO catalysts are capable of tuning of the activity and durability of Pt [10,11,12,13,14,15]. Due to the abundant resources, low price, and environmental friendliness of non-noble metal oxide components, their utility in the ORR electrocatalysis and applications in PEMFC have been extensively studied. Due to their strong interactions with Pt catalytic nanoparticles leading to the improved activity and durability during ORR, certain nanostructured metal oxides are considered as cocatalysts or active components of supports. Indeed, in the case of MO_x_-modified Pt/C hybrid catalysts, individual properties of components and synergistic interactions between them are responsible for the electrocatalytic enhancement effects. It seems that utilization of proper metal oxides permits optimization of operation of both the Pt active sites and the carbon support. Furthermore, by adding certain MO_x_ (e.g., CeO_2_, ZrO_2_, or WO_3_) formation of undesirable hydrogen peroxide has been largely suppressed due to the catalytic activity of the oxides toward H_2_O_2_ decomposition.

**Figure 1:**
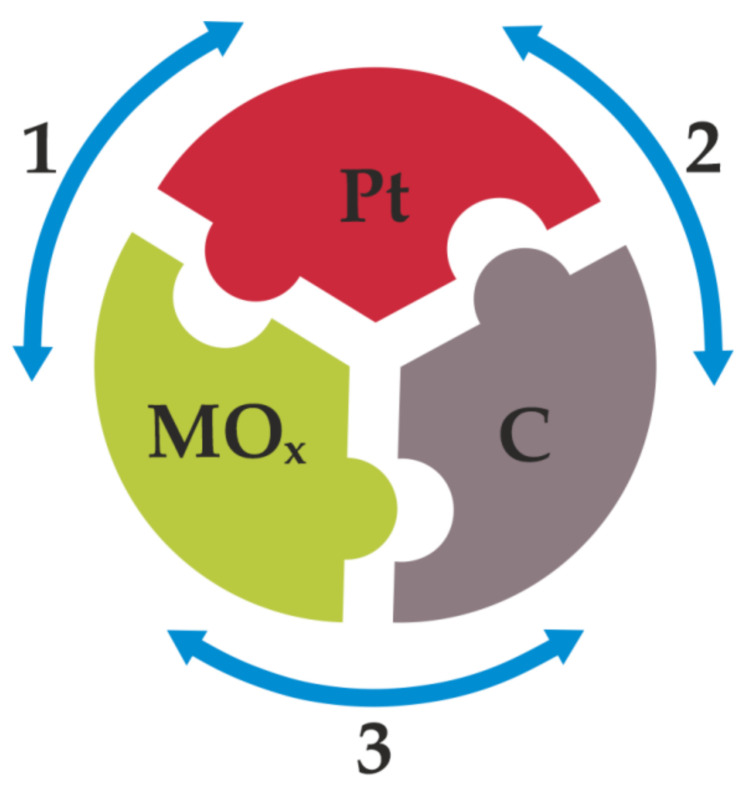
Schematic diagram emphasizing the importance of mutual interactions (marked with arrows: 1, 2, and 3, respectively) between catalytic platinum, carbon support (carrier), and metal oxide additive (cocatalyst). To explain electrocatalytic enhancement effects, we refer graphically to “the lock and key” model, explaining the specificity of enzymes. It can be rationalized that specific (activating and stabilizing) interactions should exist not only between the metal (Pt) and support (carbon carrier), but also between these components and the metal oxide cocatalyst. Likewise, enzymes, the active nanostructures, should be “flexible” and undergo some changes (e.g., structural, chemical), possibly including self-healing (e.g., through interactions with the corrosion-suppressing metal oxides).

The schematic diagram implying mutual interactions among the components of such multifunctional hybrid electrocatalysts is illustrated in Figure 1. The existence of strong interactions between MO_x_ and noble metal (Pt) nanoparticles (1) should improve the stability and activity of the metal catalytic sites due to modification of the Pt electronic structure and diminishing of the oxo (OH) species adsorption on Pt surface, thus promoting centers for the adsorption of oxygen (reactant molecule) and the cleavage of O=O bonds [16]. The interactions mentioned above would also facilitate dispersion of Pt, inhibit their detachment and further aggregation, and, consequently, prevent or decrease their degradation during the fuel cell operation [17,18].

It has been established that certain carbon nanostructures, characterized by high surface area, porosity, and high electrical conductivity, can act as suitable support materials for the deposition of catalytically active metal (Pt) centers (2). Thus, application of carbon carriers can facilitate dispersion of Pt nanoparticles, prevent their agglomeration, and may promote exposition of their specific active facets. In particular, utilization of carbons doped with different heteroatoms provide active sites for metal attachment, and tend to stabilize Pt centers at the electrocatalytic interface. Through implementation of the metal oxide additives characterized by good corrosion resistance, durability of the carbon carriers can be improved under highly aggressive (acidic) conditions existing during operation PEMFCs (3) [17,19]. Thus, it is not surprising that great deal of research has been devoted to search for suitable metal oxide materials as cocatalysts of potential importance to the PEMFC applications. Advancements in the electrocatalytic activity of platinum, in combination with the possibility of decreasing the formation of undesirable hydrogen peroxide, as well as improvement of the overall stability of both Pt nanoparticles and nanostructured carbon carriers, are crucial when it comes to the development of the low Pt content ORR catalysts.

**Figure 2:**
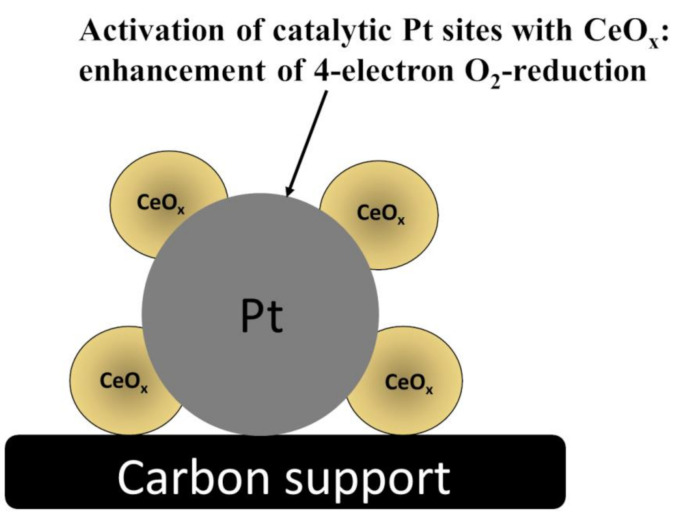
Decoration of Pt nanoparticles with CeO_x_ nanostructures. Due to the metal (Pt)—Metal oxide (CeO_x_) specific interactions, platinum is stabilized in its metallic catalytic state [22,23].

**Figure 3:**
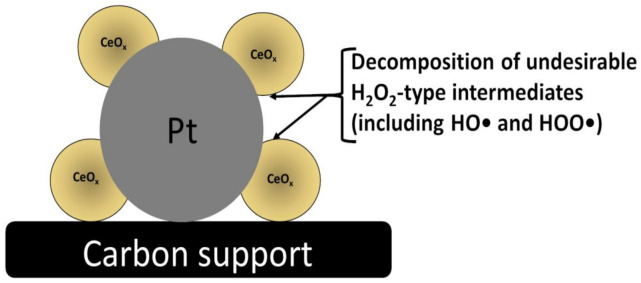
Activity of sub-stoichiometric CeO_x_ nanostructures (with Ce^III^ and Ce^IV^ ionic sites), which are existing on the catalytic platinum surfaces, toward decomposition of undesirable radicals and hydrogen peroxide intermediates [24,25,26,27].

The catalytic activity of CeO_2_ (in practice, CeO_x_, where x < 2) results from its unique structure, redox ability, oxygen vacancies, and other features resulting from the 4f electronic configuration of cerium. When it comes to ORR applications, the following features of CeO_x_ should be mentioned. The first point is associated with the presence of the oxygen defects, which are likely to serve as active oxygen adsorption sites, whereas the second feature concerns the presence of the mixed-valent Ce^III^/Ce^IV^ redox sites permitting the electron shuffling within the lattice of oxygen vacancies and enhancing the ORR activity [20,21]. The formation of oxygen-defects is accompanied by the localization of electrons left behind in Ce 4f states, thus leading to the formation of Ce^III^ species capable of elongating and reducing the O–O bond strength of the adsorbed O_2_ molecule; consequently, by increasing the relative ratio of Ce^III^-to-Ce^IV^, the ORR electrocatalytic activity can be improved [22,23]. Moreover, the increased population of Ce^III^ on the surface of CeO_2_ (or CeO_x_) particles tend to stabilize active Pt-metal centers and to improve durability of the catalytic materials (Figure 2). Our recent studies clearly show that, in the presence of ceria, the oxidative degradation of carbon carriers is also largely decreased. Furthermore, cerium oxide is known as the oxidative scavenger for free radicals such as hydroxyl (HO•) and hydroperoxyl (HOO•), which, once generated, would otherwise lead to the formation of undesirable hydrogen peroxide (Figure 3) during the two-electron oxygen reduction [24,25,26,27]. Sub-stoichiometric CeO_x_ is capable of rapidly switching between Ce^III^ and Ce^IV^ oxidation states, thus inducing the decomposition of both radicals and peroxides according to the multi-step reactions (Equations (4)–(7)):

(4) $${\mathrm{Ce}}^{\mathrm{IV}}+H_{2}O_{2}\to {\mathrm{Ce}}^{\mathrm{III}}+{\mathrm{HOO}}^{*}+H^{+}$$

(5) $${\mathrm{Ce}}^{\mathrm{IV}}+{\mathrm{HOO}}^{*}\to {\mathrm{Ce}}^{\mathrm{III}}+O_{2}+H^{+}$$

(6) $${\mathrm{Ce}}^{\mathrm{III}}+{\mathrm{HO}}^{*}+H^{+}\to {\mathrm{Ce}}^{\mathrm{IV}}+H_{2}O$$

(7) $${\mathrm{Ce}}^{\mathrm{III}}+{\mathrm{HOO}}^{*}+H^{+}\to {\mathrm{Ce}}^{\mathrm{IV}}+H_{2}O_{2}$$

**Figure 4:**
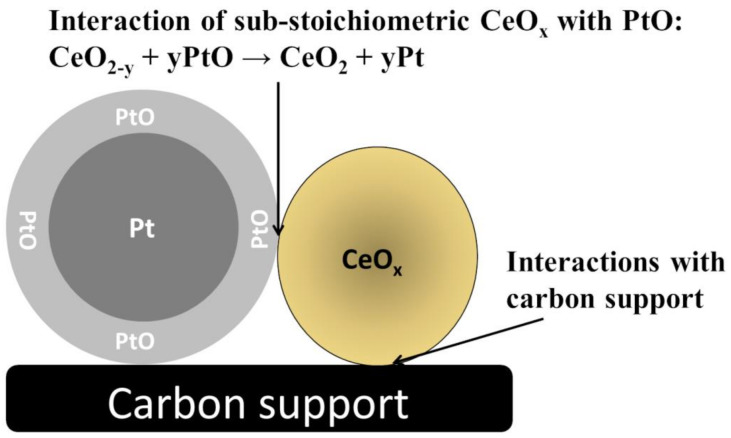
Importance of specific interactions of substoichiometric CeO_x_ (having Ce^III^ and Ce^IV^ ionic sites) with platinum oxo-species (diminishing formation of PtO or PtOH on catalytic Pt [29,32]) and carbon support (suppressing its corrosion).

The importance of the presence and distribution of Ce^III^ and Ce^IV^ ionic sites should be mentioned here. Larger populations of surface Ce^III^ species facilitate scavenging of hydroxyl radicals (Equations (6) and (7)); on the other hand, Ce^IV^ species tend to induce decomposition of hydrogen peroxide (Equations (4) and (5)). These reactive oxygen species are highly corrosive, and are commonly recognized as initiators of the degradation of proton exchange membranes [28]. In this respect, introduction of such metal oxides as CeO_x_ to the low-Pt-content electrocatalytic interfaces seems to be of primary importance, as the latter systems suffer from the problem of generating high amounts of the undesirable hydrogen peroxide intermediate. In view of the above, considerable attention has been paid to the investigation of Pt-CeO_2_ hybrid electrocatalysts for ORR in PEMFCs [14,29,30,31]. When the ORR enhancement effect originating from the presence of CeO_x_ has been addressed by performing in situ EXAFS measurements [32], the importance of the boundary formed between the Pt-metal and CeO_x_-metal oxide has been emphasized [29,32] and explained in terms of the inhibition of Pt oxide formation by the CeO_x_ layer (Figure 4). The above observation seems to be very helpful when it comes to designing highly active and durable ORR catalysts containing low platinum loadings [11,14].

A facile one-pot synthesis procedure for the fabrication of CeO_2_ nanoparticles (deposited on carbon support) with high concentration of oxygen vacancies (acting as the nucleation sites for Pt nanoparticles) was proposed [14]. The obtained Pt-based catalysts which contained 10–30 wt% CeO_2_ were characterized by more positive half-wave oxygen reduction potential, *E*_1/2_ = 0.86 V, and higher mass activity (at 0.90 V) in the range from 39.54 to 52.09 mA mg^−1^ relative to the analogous parameter determined for the conventional carbon (Vulcan) supported platinum, Pt/C: *E*_1/2_ = 0.85 V vs. RHE, and mass activity, 36.44 mA mg^−1^. Moreover, the electrochemically active surface areas were found to be higher for all CeO_x_ modified (admixed) catalytic systems. The latter observation was attributed to better morphology (smaller sizes of Pt centers) and more homogeneous distribution of catalytic platinum sites. The accelerated durability test (performed in the range of 0.6–1.2 V vs. RHE) showed a fivefold enhancement of stability of the Pt/CeO_2_ composite catalysts, in comparison to the pristine Pt/C.

The preparation of highly active triple-junction-interface composed of Pt and CeO_2_ nanostructures, as well as carbon nanotubes, CNTs, could also be achieved by atomic layer deposition [11]. It was then rationalized that the system, in which Pt had contact with carbon materials and metal oxides, could improve dispersion of the platinum catalytic sites. In addition, the presence of oxygen vacancies in the CeO_x_ component facilitated shifting the ORR potential toward more positive values, the appearance of larger ORR current densities, as well as the decrease in the hydrogen peroxide intermediate formation. On the basis of the XPS data, the variation of the electronic structure of platinum, the content of Ce^III^, and the population of oxygen vacancies were rationalized in terms of stronger interfacial interactions between Pt and CeO_2_ in the presence and absence of CNTs. Moreover, the application of the atomic layer deposition method allowed the synthesis of highly and uniformly dispersed Pt nanoparticles characterized by high ECSA values. In this respect, the combination of sub-stoichiometric CeO_x_ and highly conductive CNTs, with respect to the enhancement of the Pt activity toward ORR, should be emphasized. Furthermore, the obtained catalysts showed a decrease of about 13% in the mass activity after 5000 cycles, whereas the commercial Pt/C was characterized by 46% loss, under analogous conditions [11]. The enhanced durability and better corrosion resistivity of Pt-based electrocatalysts utilizing carbon nanotubes and cerium oxide, relative to commonly used Vulcan XC-72 support, was also confirmed by others [33]. These features are of importance when it comes to the preparation of low-Pt-content catalytic systems. It is reasonable to expect that application of CNTs should also facilitate charge distribution to the dispersed Pt catalytic sites and improve the overall conductivity at the electrocatalytic interface.

The enhanced catalytic activity towards ORR and stability of Pt-CeO_x_ nanocomposite supported on CNTs has also been demonstrated when a sacrificial precursor based on a metal-organic framework is used [34]. In this respect, the application of the cerium-containing metal-organic frameworks allowed the preparation of unique electrocatalysts in which CeO_x_ was intimately connected to the surfaces of highly dispersed Pt nanoparticles. Furthermore, it was rationalized that CeO_x_, derived from the metal-organic framework, tended to stabilize metallic platinum sites in the catalytic nanocomposite. A beneficial role of the defective structure of ceria was also emphasized in the context of strong Pt-CeO_x_ interactions, resulting in favorable electron movement from the CeO_x_, and filling the d-band vacancies in the Pt nanoparticles. It was also suggested that the lowering of the ORR activation barrier could be attributed to the oxygen spillover and preferential adsorption of OH groups on the oxide, rather than Pt surfaces. However, even if ceria species could undergo dissolution at the interface formed with the polymer electrolyte layer in PEMFC, they would act as radical scavengers to decrease the membrane degradation [35]. Nevertheless, more advanced research on the selectivity, performance, and reaction pathways and origin of enhancement effects at Pt-CeO_x_ type electrocatalysts is still needed, particularly if the efficient low-Pt-loading systems are going to be designed and successfully utilized.

Due to excellent chemical stability in acidic solutions and intrinsic catalytic activity observed in various applications, niobium oxide nanoparticles were investigated as components of ORR catalysts [13,16,36,37,38,39]. The influence of various forms of niobium oxide on activity low-Pt content electrocatalysts was also addressed [16]. It was found that during ORR the carbon-supported hybrid systems composed of Pt and NbO_2_, Pt-NbO_2_/C, were characterized by the approximately three times higher mass and specific activities, in comparison to the performance of the commercial Pt/C electrocatalyst. Using the rotating ring-disk voltametric methodology, the four-electron ORR mechanism was determined in despite of very low Pt loadings, namely on the level of 5 µg cm^–2^. Furthermore, the improved stability of Pt-NbO_2_/C was also demonstrated. Following application of 30,000 potential cycles from 0.6 to 1.1 V (vs. RHE) to the sample in an O_2_ saturated solution, the system’s half-wave potential declined about 23 mV, whereas the commercial Pt electrocatalyst showed a negative shift of 40 mV and an approximately 45% loss in the Pt surface area. The better activity and stability of Pt-NbO_2_/C were explained in terms of strong interactions between platinum and niobium oxides that decreased OH-adsorption on Pt surfaces due to the existence of lateral repulsion between PtOH and the niobium oxide surface species. In addition, the possible impact of surface vacancies existing within the niobium oxides was also highlighted. Some dissimilarities in activities between samples containing two distinct niobium oxide nanostructures were described and correlated with the differences in their electronic structures and electrical conductivities [13]. A sample utilizing the NbO suboxide showed enhanced ORR activity in acidic media with the characteristic onset potential on the level 1.0 V, and a half-wave potential equal to 0.86 V. The improvement in electrocatalytic properties, when compared to the niobium-oxide-free Pt/CNT or commercial Pt/C catalysts, was interpreted in terms of the reduced OH adsorption on nanostructured Pt surfaces in the presence of niobium oxide nanostructures. Such features as suppression of the dissolution of Pt, surrounded by robust niobium oxide, as well as feasibility of specific metal–metal oxide activating interactions should also be mentioned here. Indeed, the Pt-NbO/CNT system exhibited excellent stability during diagnostic tests. Even, upon introduction to PEMFC at the loading as low as 0.15 mg cm^–2^, the Pt-NbO/CNT catalytic system shows very good performance with the maximum power density of 772 mW cm^–2^ and only 4% decline of power after 96 consecutive hours of operation at 80 °C.

Keeping in mind that interactions between the metal (Pt) active centers and metal oxide support component (cocatalyst) play a crucial role in the overall catalytic activity and stability, the interface between Pt and NbO_x_ has been addressed [40]. The Pt-NbO_x_/C catalytic system with tunable structural and electronic properties has been prepared using the modified arc plasma deposition method. Although the degree and exact nature of interactions between the metal (Pt) and the oxide are dependent on the physicochemical identity of niobium oxide, it has been postulated that electrons are generally shifted from Pt to NbO_x_. Under such conditions, the Pt-Pt bond is shortened at the electron depleted surfaces of platinum nanoparticles (surrounded by the nearly electron-saturated NbO_x_ nanostructures) and, as a result of activating interactions with O atoms of O_2_ molecule, the double O=O bond is weakened, which facilitates its dissociation and improves the ORR performance. In comparison to the conventional Pt-C system, the Pt-electron-donation trend has been more pronounced in the presence of niobium oxide, what translates to larger electron deficiency at the interface capable of stabilizing low-coordinated Pt sites and facilitating dispersion of small metal (Pt) nanoparticles and their high catalytic durability.

Considerable attention has also been dedicated to materials based on titanium oxides as durable catalytic supports for PEMFC applications due to their inherent stability under various electrochemical conditions, high corrosion resistance, and enhancement of electrocatalytic activity through the bilateral interactions effect originating from the combination of catalytic metal nanoparticles and TiO_x_ [41,42,43,44]. It has been reported that TiO_2_ prevents agglomeration of Pt nanoparticles and effectively facilitates the dispersion of Pt sites in the robust metal oxide clusters [45]. Furthermore, TiO_2_ supports not only control and facilitate nanostructuring of the catalyst, but also provide both thermal and oxidative stability. However, the low electrical conductivity of titanium oxide supports or cocatalysts attenuates the observed electrocatalytic enhancement effects, which becomes a considerable disadvantage for ORR applications. While having band gaps on the level of 3.0 and 3.2 eV, respectively [46], both the rutile and anatase forms of TiO_2_ are semiconductors. Among ideas to improve the electronic conductivity of TiO_2_, introduction of appropriate dopants or addition of conducting components, such as carbons, are the most promising approaches. For example, it has been reported [47] that the TiO_2_-CNT-supported Pt catalyst exhibited very good performance during ORR, largely due to high conductivity of the CNT additives. On the contrary, the carbon-carrier-free Pt-TiO_2_ system has exhibited the lower ORR activity due to the limited macroscopic electronic conductivity of titanium oxide. The Pt-TiO_2_/CSCNT system (where CSCNT stands for cup-stacked carbon nanotubes) has been characterized by high ORR activity, the selectivity towards 4-electron oxygen reduction, and by the improved stability which may be explained in terms of strong metal–support interactions between Pt nanoparticles and TiO_2_ [41]. In the case of the Pt nanoparticles deposited onto the TiO_2_/CSCNT, the XPS results suggested local increases in the electron densities on Pt surfaces, which have been interpreted in terms of the donation of electrons from the TiO_2_ to platinum sites. Moreover, the obtained electrocatalyst was characterized by limited tendency of platinum nanoparticles to undergo agglomeration and, consequently, it exhibited long-term durability.

An interesting case involves the combination of the catalytic metal (Pt) and the functionalized oxide (Nb-TiO_2_) support [48]. The analysis, based on density functional theory (DFT) calculations, implies that, following the introduction of platinum onto Nb-doped TiO_2_, the d-band center of Pt has been decreased, and its electronic structure has been altered. Thus, weakening of the bonds in the adsorbed oxygen molecule can be envisioned according to the d-band theory [49]. Consequently, a decrease in the ORR overpotential has been postulated for such catalytic systems. In another example, TiO_2_ can also be utilized as supplementary activating component for Pt catalytic nanoparticles supported onto multi-walled CNTs [50]. Electrochemical tests revealed higher ORR activity and better durability of Pt-TiO_2_/CNT systems, when compared to that of the commercial Pt/C. In spite of semiconducting properties of TiO_2_ additives, conductivity on multi-walled CNT carriers should be particularly advantageous when it comes to designing the low-Pt-content systems. Functionalization, derivatization, or admixing of the metal oxide additives seems to be an important direction for future research aiming at the improvement of activity and stability of the Pt-containing electrocatalytic systems for ORR.

Moreover, it has been demonstrated that, by increasing the loading of TiO_2_, the durability of the Pt-TiO_2_/CNT catalyst can be improved without affecting the system’s ORR activity. However, care must be exercised to minimize Ohmic limitations and assure unimpeded charge propagation at the electrocatalytic interface. Among the possibilities to improve the electronic conductivity of titania is the thermal treatment of TiO_2_ in the reducing environment, leading to the formation of substoichiometric Magnéli phases (Ti_x_O_2x−1_) having oxygen vacancies in the titania crystalline lattice and exhibiting graphite-like conductivity [44,51]. It has been postulated that Ti^III^ defects or oxygen-vacancies in titanium sub-stoichiometric oxides can act as charge electron recombination sites facilitating stable performance of the Pt-containing catalytic system during ORR [12,51,52]. Doping with transition metals is another promising approach to obtain a defective non-stoichiometric titanium oxide support components [48,53,54,55,56] but further research is needed along this line.

**Figure 5:**
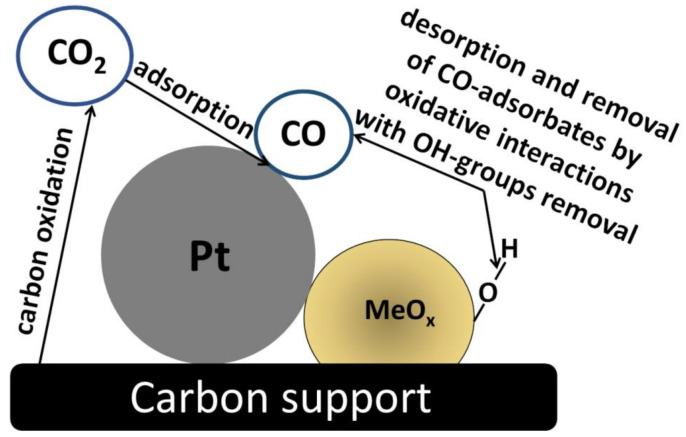
Cartoon illustrating interactions of hydroxyl groups on the metal oxide, MeOx (e.g., ZrO_2_, CeOx, and TiO_2_) with CO adsorbates existing on platinum (as a result of the oxidation/corrosion of carbon supports) leading to the removal of CO-poisoning species.

Among other metal oxide support materials or additives, ZrO_2_ also requires some attention due to its excellent thermal stability and resistance to oxidative degradation and acid corrosion, namely the features that can effectively improve the durability of catalysts [57]. Furthermore, it was found that the zirconia-containing ORR catalysts were characterized by high tolerance with respect to poisoning with organic molecules, such as methanol [58]. On the other hand, practical utilization of ZrO_2_ nanostructures in fuel cell technology is limited by the oxide’s low electrical conductivity. To overcome this problem and facilitate distribution of electrons at ZrO_2_-containing interfaces, an important strategy involves application of nitrogen-doped carbons together with ZrO_2_ nanostructures [59]. It is apparent from the diagnostic electrochemical experiments that Pt nanoparticles, which are well-dispersed on such supports, exhibit higher activity during ORR, when compared to the performance of those without zirconium oxide. In addition, it has been demonstrated that the application of protective ZrO_2_ nano-shells (nano-deposits) on surfaces of carbon carriers can solve the well-known problem of the oxidative corrosion of carbon supports, e.g., leading to the formation of CO species (Figure 5) during ORR [60]. In addition to chemical interactions permitting the removal of poisoning CO adsorbates (Figure 5), the ZrO_2_ over-layers existing on carbons minimize their direct physical contact with the acid environment. Furthermore, due to specific interactions of Pt with ZrO_2_, migration and aggregation of Pt nanoparticles are largely prevented [61]. It should also be remembered that decomposition of the hydrogen peroxide undesirable intermediate is induced in the presence of zirconia [62].

In the search for suitable metal oxide nanomaterials (such as additives and cocatalysts) for decoration of carbon carriers and for supporting catalytic platinum nanoparticles, tungsten oxide (WO_3_) and tantalum oxide (Ta_2_O_5_) were also considered [63,64,65]. The increased specific activity of the resulting hybrid systems toward the ORR was explained in terms of d-band approach explored together with density functional theory. It was rationalized that position of the d-band center of platinum should be brought down in the presence of tantalum(V) oxide, which would result in the weakening of the bonds existing between platinum and adsorbed oxygen species. Furthermore, the preferential adsorption of -OH groups on surfaces of the oxide, rather than catalytic platinum, should favor the metal’s catalytic activity. The bifunctional activity of the tungsten oxide based system in terms of promoting both reduction in oxygen (at traces of Pt) and reductive decomposition of the H_2_O_2_ intermediate (at mixed-valent W(VI,V)) was also postulated. In general, this supports utilizing partially reduced WO_3_ [64], in which hydrogen tungsten(VI,V) oxide bronzes, H_x_WO_3_, coexisting with substoichiometric oxygen-deficient tungsten(VI,IV) oxides, WO_3−y_ (0 < y < 1), were characterized by fast electron transfers, good proton mobility, and high porosity, as well as by high reactivity toward reductions in such inert reactants as oxohalogenates and hydrogen peroxide.

## 3. Alloyed Pt Nanostructures

A reasonable approach which may lead to a reduction in the level of Pt utilization is to consider the partial replacement of Pt with other (preferably non-noble) metals, provided that the obtained catalytic system is not only less expensive, but also more efficient during ORR. The electrocatalytic properties of bi- or multi-metallic Pt-containing alloys reflect combinations of the features of individual metal components, as well as the possible synergistic interactions between them [66]. The improved catalytic activities, relative to those characteristic of the bare (pure) Pt metal, have been demonstrated using the combination of Pt with different metals, such as Pd, Au, Ag, Ni, Co, Cu, and Fe [4,6,67,68,69,70,71,72,73,74,75]. The observed enhanced electrocatalytic activities have been attributed to geometric (decrease in the Pt–Pt bond distances) and electronic (increase in the d-electron vacancy in Pt) factors, in addition to the interfacial structural changes affecting the adsorption (O_2_-molecule-activation) properties [76,77,78]. Indeed, the binding energy of O_2_-adsorbate to the metal surface reflects the electronic structure of the surface itself. The coupling between the oxygen-2p-states and the metal d-states leads to the generation of bonding and antibonding states, for which the extent of filling depends on the local electronic structure of the electrocatalytic interface and their positions relative to the Fermi level [79]. The parameter, which is correlated with the extent of filling of the antibonding molecular orbital, is the location of the d-band center [80]. An upward shift of the d-band center with respect to the Fermi level corresponds to an increase in energy and decrease in the degree of filling of the antibonding states, thus leading to a stronger bonding between the metal and the adsorbate [79]. On the basis of relationships [76] between specific activities of Pt_3_M bimetallic alloys (monitored at 0.9 V vs. RHE during ORR in 0.1 mol dm^−3^ HClO_4_ at 333 K) and the d-band center positions characteristic of the Pt-skin and Pt-skeleton surfaces, it has been postulated that the maximum catalytic activities of Pt_3_M (M = Ni, Co, Fe, Ti, or V) alloyed catalytic systems are dependent on the balance between adsorption energies of the ORR reactive intermediates and surface coverages of the blocking species [76].

Application of Pt-alloyed catalytic systems permit reducing the amount of Pt by improving the mass activity during ORR, at least in comparison to the conventional Pt/C. However, under the fuel cell operating conditions, their practical long-term durability is still insufficient and constitutes one of the challenges to be addressed in the near future. The problem is related to the dissolution of the second metal (e.g., Co, Fe, Ni) in Pt-alloys and transfer into the PEM membrane electrode assembly (MEA) of the fuel cell, thus resulting in significant performance losses. The degree of degradation of bimetallic alloyed catalysts depends on both nature of the second (added) metal and the atomic ratio (relative to Pt) [81,82,83]. In addition to the need of the composition optimization, the control of morphology and shapes of the alloyed catalytic systems is as important as in the case of the monometallic catalysts, in the context of their activity and stability.

Among Pt-bimetallic alloys, the bimetallic PtNi nanomaterials have been demonstrated as the most promising ORR catalytic materials [66,69,84,85,86]. In particular, the octahedral PtNi nanoparticles with well-extended (111) facets exhibit the promising ORR activity, namely superior in comparison to performance of the Pt(111) counterpart and the commercial Pt/C catalysts [66,69,87]. For instance, despite the low platinum content (2.76 wt%), and the high activity of Pt_0.61_Ni/C cathode catalyst, reaching the maximum power density of 1.1 W cm^−2^ (at 80 °C) has been reported during operation of low-temperature hydrogen-oxygen fuel cells [84]. In practice, there is a need to increase the stability of PtNi nanomaterials [88]. This necessity can be achieved by introducing a third element to form a ternary alloy or by modification through surface doping [89,90,91,92,93,94,95]. The enhanced durability and activity of PtNiCu nanoparticles, relative to octahedral PtNi [91], seems to reflect the surface elemental distribution of metal components. Based on the theoretical and experimental data, the increased population of the surface Pt atoms has been postulated in such ternary alloys. Under such conditions, the interfacial dissolution of the transition metal additives (Ni and Cu) is diminished, as well as formation of surface vacancies suppressing dissolution of atoms from sub-surface layers is feasible. It has also been reported [89] that incorporation of gallium into PtNi nanoparticles can modify their electronic structure and stabilize the Ni-component, even under acid conditions. Relative to PtNi/C and the conventional Pt/C, the superior ORR-performance of Ga−PtNi/C, both during half-cell and single-cell tests, has been attributed to changes in the binding energies of the ORR intermediates, the reduced oxyphilic surface, and the Pt-lattice compression induced by Ga doping. The beneficial role of the doping of PtNi/C with Mo (mostly in the form of oxides) has also been reported [90,93,96]. The fact that molybdenum surface dopant preferentially occupies the vertex and edge sites of Mo-PtNi/C results in stabilization of the desired octahedral morphology of PtNi (enriched with exposed (111) facets in acid environment) as well as the increase in concentration of the sub-surface Ni and the stabilized under-coordinated Pt sites, thus preventing their migration and dissolution. In addition, the high Ni retention within the PtNi alloy phase and presence of molybdenum oxides at the interface are likely to tune the absorption or desorption energies of the oxygen-containing species, thus inducing the ORR dynamics [90,93].

The PtCo alloys are also promising electrocatalysts for ORR with the demonstrated practical utility in the fuel cell devices [72,78,97,98,99]. As before, the major issues concern the enhancement stability of PtCo catalysts as well as substantial reduction in the platinum loading during practical applications. Due to the existence of synergistic interactions between Pt and platinum-group-metal (PGM) free catalysts [100], the content of platinum could be substantially reduced while maintaining excellent activity and durability. Therefore, the concept of fabrication of the catalytic systems utilizing the ultralow-loading of the platinum-cobalt catalyst dispersed over PGM-free materials has been pursued. While using the Co-containing or the Co- and Zn-containing zeolitic imidazolate frameworks (ZIFs) as the precursors, three different electrocatalytically active forms, bimetallic Pt-Co, Co-N_x_-C_y_, and Co, encapsulated within onion-like graphitic layers, have been obtained. The outstanding ORR activity and high stability have been postulated. The increased PtCo alloy efficiency has been attributed to strong binding of PtCo nanoparticles to PGM-free site-mediated surfaces, enhancements in the charge distribution and the reaction intermediate transfers, as well as the decrease in the formation of H_2_O_2_ generated at nearby PGM-free sites, thus promoting the effectively almost-four-electron ORR mechanism. The stabilization of PtCo alloys with the C- and N-ligands has also been postulated [101]. Here, the performance of PtCo “core-shell” carbon nitride-based electrocatalysts has been found to be dependent on the structure, composition, and nature of interactions with the nanocomposite zeolitic inorganic–organic polymer electrolyte precursors. Typically, the decrease in mass-activity (measured at 0.9 V) of such catalysts (following the accelerated stability tests involving 30 000 voltametric potential cycles between 0.6 and 1.0 V) has been found to be less pronounced in comparison to the behavior of conventional Pt/C [99]. Overall, the improved performance and stability of the PtCo “core-shell” carbon nitride-based electrocatalysts has been credited to the formation of an intermetallic structure that mitigates oxidation of the catalytic Pt and dissolution of the Co-bimetallic-component.

Application of Au clusters constitutes another approach to stabilize the underlying Pt metal surfaces under highly oxidizing conditions and to suppress Pt dissolution without decreasing the ORR dynamics [102]. Based on the data from in situ X-ray Absorption Near-Edge Spectroscopy (XANES) studies, it has been postulated that interfacial oxidation of Pt nanoparticles covered by Au is largely decreased. Highly stable and active ORR electrocatalysts composed of platinum monolayers on Pd_9_Au_1_ alloys have been proposed [70]. Indeed, the proposed Au-containing materials have exhibited only a few percent decrease in activity over 100,000 voltametric cycles in the potential range from 0.6 and 1.0 V. The improved stability of the platinum monolayer on Pd_9_Au_1_/C results from the inhibition of the Pd oxidation and dissolution (corrosion) as a consequence of shifting the Pd oxidation potential by alloying Pd with Au. Interactions between the two metal components and changes in the electronic structure of the PdAu system have also been suggested [103].

## 4. Heteroatom Doped Carbon Carriers

**Figure 6:**
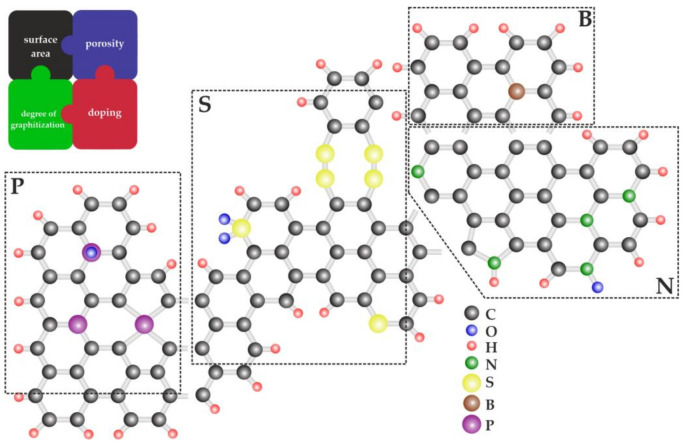
Cartoon illustrating the concept of doping of carbons with heteroatoms resulting in the presence of surface functional groups, as well as emphasizing the importance of carbon porosity, surface area, and the degree of graphitization.

In general, carbons are characterized by high electrical conductivity, and large surface areas are used as supports for Pt-based catalysts. As illustrated in Figure 6, among other important factors are the carbon porosity, degree of graphitization, and the presence of surface functional groups [17,104,105,106]. Due to insufficient resistance against the oxidative corrosion of the commonly used carbon blacks (Vulcan XC-72 and Ketjen black), the recent fuel cell research has concentrated on functionalization and modification of carbon materials to mitigate this limitation [107]. The sensitivity of carbon blacks toward degradation under harsh oxidative conditions should be correlated with the presence of highly amorphous carbon arrangements and the absence of long-range ordered graphitic structures. Insufficient durability of carbon supports (carriers) results in the undesirable detachment and agglomeration of Pt nanoparticles, thus leading to the loss of the electrocatalytic active surface area and, consequently, the decreased system’s performance [107,108,109]. Moreover, the relatively weak Pt–support interactions could increase the overall electron-transfer resistance at the interface, and impose the additional ORR overpotential. The right balance between the sufficiently high degree of graphitization and certain surface properties of carbons, such as adequate surface area and porosity, could assure acceptable stability while providing the proper catalytic enhancement effects [110]. Existence of the porous structure of carbon is crucial to the dispersion of Pt catalyst and to its better utilization at the interface formed with ionomers. Furthermore, the carbon porosity facilitates mass transport during ORR and boosts the MEA performance. It has also been found that, by increasing the support’s porosity, migration and coalescence of the Pt catalytic particles are decreased and, consequently, the retention of electrochemical surface area (ECSA) is improved [111]. The interfacial interactions between the Pt-metal active sites and the carbon matrix are of importance as well. Doping carbons with different heteroatoms, such as sulfur, nitrogen, phosphorus, and boron (Figure 6), could result in increased surface hydrophilicity and polarity, changes of the acid-base properties, and formation of active centers for adsorption and driving the catalytic ORR [112].

The concept of doping with nitrogen, which is capable of not only increasing electric conductivity but also catalytic activity of carbon materials, has been so far the most extensively explored [112,113]. The enhanced activity of the nitrogen-doped materials is usually correlated with the incorporation of the more electronegative N (3.04) atoms, relative to C (2.55) atoms within the sp^2^ carbon lattice, thus a different charge density among the adjacent carbon atoms, and facilitating the oxygen chemisorption [114,115]. For example, it has been demonstrated that N-doped reduced graphene oxide provides suitable support for anchoring Pt nanoparticles of 2.8 nm diameters [116]. The resulting hybrid reduced-graphene-oxide-supported low-Pt-content (5.31 wt%) catalyst has exhibited high ORR electrocatalytic activity and very good stability which have been attributed to the existence of the synergistic effect between N-doped carbon matrix and Pt, as well as to the presence of the stable Pt-N-C chemical bonds. The doping nitrogens have been reported to provide pathways for the distribution of electrons at the electrocatalytic interface as well as to act as bridges between the support and Pt nanoparticles, thus preventing the Pt-metal active sites from detachment, dissolution, migration, and aggregation. In particular, the enhanced electrocatalytic performance of Pt deposited onto N-doped graphene nanosheets has been attributed to interfacial changes of the platinum electronic structure in the presence of nitrogen functionalities and to better utilization of Pt catalytic sites during ORR [117]. The effect of electronic interactions between Pt and N-doped carbon on the ORR performance has been studied and analyzed using XPS and DFT approaches [118]. Here, the formation of Pt-N chemical bonds has been postulated, which facilitates electronic transfers from Pt sites to the carbon support and induces changes in the oxygen adsorption energy at the platinum surface. This strong interaction led to ca. about 2.1-fold increase (relative to the performance of Vulcan XC-72 support) in the ORR mass activity at 0.9 V vs. RHE. The weaker adsorption of molecular and atomic oxygens has also been demonstrated in the case of the N-doped substrates and correlated with the elongated O-O distance and lower energetic barrier for the O_2_ dissociation. In addition, the performed calculations have implied strong interactions between Pt clusters and N-doped graphene, thus leading to lower resistance for electron transfers facilitating electrochemical reactions and improving the stability of the catalyst. The improved ORR performance and higher mass activities of Pt electrocatalysts deposited on N-doped carbon supports have also been attributed to beneficial platinum dispersion, in addition to strong metal–support interactions [119,120,121,122,123]. 

While the N-doped carbons have become the most widely explored systems, the doping of carbons with sulfur or boron also seems to be promising for electrocatalytic ORR [113]. As a sulfur atom is somewhat larger, its incorporation into the carbon lattice induces strain and stress, but it may allow for a targeted tuning of the carbon band gap, depending on the number of the incorporated sulfur atoms [124,125]. Moreover, the DFT calculations have demonstrated that the spin effect, induced by S atom incorporated into carbon network, can tune the *OOH binding energy and facilitate the oxygen adsorption and the O–O bond cleavage [126]. The ORR electrocatalysts, which are characterized by the improved activity and durability through modification of the activity of Pt nanoparticles by interactions with S-doped carbon layers, have also been synthesized [127]. It has been rationalized that the exposure of platinum to sulfur species facilitate the nucleation of Pt nanoparticles and induce their uniform distribution. In the presence of the S-doped carbon functional layer, the O–O dissociation step of the ORR mechanism seems to be favored by lowering the *OOH coverage on Pt. Additionally, the boron doping of the carbon supports provides more sites for noble metal anchoring, generation of Pt particles of small sizes, and better dispersion of the active sites [128,129]. Based on the density functional theory calculations, it is reasonable to expect significant increases in the adsorption energies for Pt in the presence of boron originating from the B-doped carbon supports. From a fundamental point of view, strong hybridization and admixing of the Pt d-orbital and the B p-orbital, leading to the formation of direct chemical bonding between Pt and B atoms, can be expected [130,131]. Enhancement of the activity of Pt deposited on B-doped carbon toward ORR has also been attributed to the electronic effects facilitating the O_2_ adsorption on Pt surface. Better corrosion resistance of the B-doped carbon support, which improves the system’s overall stability, has also been reported [129]. 

Using phosphorus as a doping element for carbon support materials seems to be a promising strategy to improve the long-term stability of the ORR electrocatalysts due to the ability of P to inhibit the oxidative carbon corrosion processes [132,133]. Furthermore, phosphorous functional groups can effectively modify the electrical properties and chemical reactivity of the carbon matrix, thus making them attractive to numerous applications, especially in catalysis [134,135]. For example, the low Pt content electrocatalysts deposited on phosphorus-doped carbon nanotubes have been found to exhibit the enhanced ORR activity and long-term stability in acidic media [136]. The improved electrocatalytic performance is the result of the strong interactions between Pt and P-decorated CNTs, which has been confirmed by X-ray photoelectron spectroscopic analysis and density functional theory calculations. Furthermore, the interfacial changes in electron densities on CNT supports containing P-atoms of donor electron properties has been postulated. Moreover, platinum deposited on such supports exhibits better tolerance to methanol crossover when compared to the behavior of the conventional Pt/C.

## 5. Conclusions and Perspectives

Reactivity of low-Pt-content catalytic materials for oxygen reduction largely depends on the choice of carbon (or other) support, its modification or functionalization, as well as addition of cocatalytic active sites or components. With the present state-of-the-art, typical ORR catalysts are still carbon-supported Pt based systems. Keping in mind the lack of stability of carbon materials in the fuel cell acid environments, the currently used carbon supports (such as Vulcan XC-72R and Ketjen) do not meet durability requirements for many (including automotive) applications. Practical supports should be characterized by excellent electronic conductivity, high corrosion resistivity, uniform distribution of Pt nanoparticles, high surface area, and strong (but not inhibiting) interactions with Pt centers. Of particular interest are hybrid systems containing nanostructured metal oxides, platinum alloys, or various functionalized carbon carriers, in which the lattice carbons are replaced with heteroatoms (e.g., N-, P-, B-, or S-doped). Interaction of such surface sites with Pt sites would often lead to enhancement of the electrocatalytic activity. The use of derivatized or functionalized carbon supports can not only reduce the amount of the precious Pt catalyst, but also enhance catalytic activity and stability. Additionally, certain sub-stoichiometric metal oxides (e.g., CeO_x_, NbO_x_, TiO_x_, and ZrO_x_) with interfacial defects can be successfully used as the components of supports, and they are capable of interacting (activation, stabilization) with both Pt centers and carbon carriers, in addition to the feasibility of anchoring noble metal nanoparticles (e.g., Pt or bimetallic alloys) for ORR in acid media. Overall, the 3d-metal supported or alloyed Pt electrocatalysts perform better than pure Pt as the surface electronic structure of platinum coexisting with the metal is somewhat modified.

It is reasonable to expect that the prospective catalytic systems will utilize distinct components, e.g., heteroatom doped carbon carriers (or their combinations), unmodified or decorated with metal oxide cocatalysts or stabilizers, alloyed (with one or two transition metals) Pt-based catalytic centers at the optimized ratios and chemical identities. With ongoing progress in the area of nanostructure engineering, fabrication and utilization of catalytic mixed metal oxides may lead to further improvements in the performance of the ORR catalysts in terms of their conductivity, activity, and stability.

More attention would have to be paid to requirements coming from the reaction pathways, i.e., to the mechanistic and kinetic ORR details. Formation of hydrogen peroxide type intermediates would have to be further decreased in practical, particularly low-Pt-content systems. Once more, the low-Pt-content systems are likely to be multi-component and multi-functional, with distinct catalytic sites, including those capable of inducing decomposition of H_2_O_2_. However, care should be exercised with respect to the degree of nanostructuring of the catalytic components. Almost all truly nanostructured materials have rather low conductivity, exhibit higher solubility in aqueous environments (water is the reaction product during operation of the hydrogen-oxygen fuel cell), and often are subject to the oxidative degradation. Thus, there is still a need to develop synthetic methodology, and properly modify existing or fabricate new support materials which are characterized by high surface areas and have improved electronic conductivity, insolubility, and stability. Another issue concerns the progress in the deposition of Pt or Pt-alloy nanoparticles on new or modified supports, with the need of creating and understanding the activating metal–support interactions, as well as taking into account stability issues. On practical grounds, it would be difficult to obtain the ideal ORR catalytic material capable of meeting all expectations discussed above, but some realistic compromise seems to be possible.
